# Supplementary material for: Skin muscle is the initial site of viral replication for arboviral bunyavirus infection
Source: Nat Commun. 2024 Feb 6;15:1121. doi: 10.1038/s41467-024-45304-0 (PMC10847502; doi:10.1038/s41467-024-45304-0)
Supplement: Supplementary file 1 — Supplementary Information [file 41467_2024_45304_MOESM1_ESM.pdf]

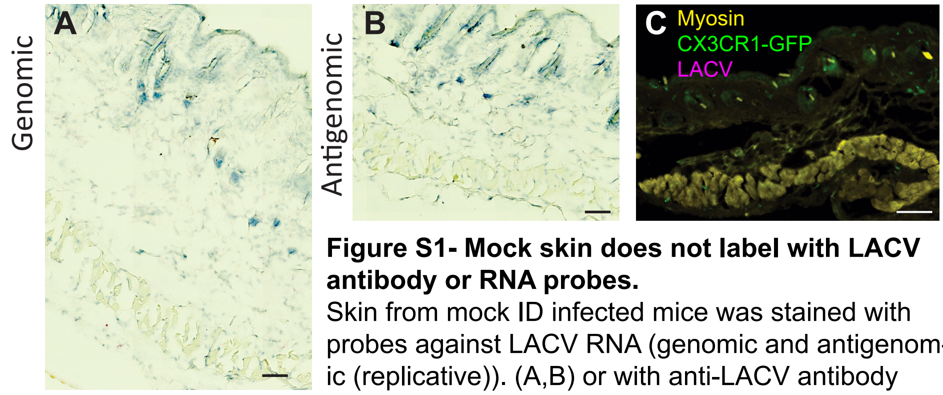

Figure S1- Mock skin does not label with LACV antibody or RNA probes.
Skin from mock ID infected mice was stained with probes against LACV RNA (genomic and antigenomic (replicative)). (A,B) or with anti-LACV antibody (magenta) (C). For (C), skin was also stained with anti-myosin (yellow) and anti-GFP (green). All images are n = 2 mice, with 2 sections of skin per mouse stained in independent experiments with similar results. Scale bars represent 50 µm

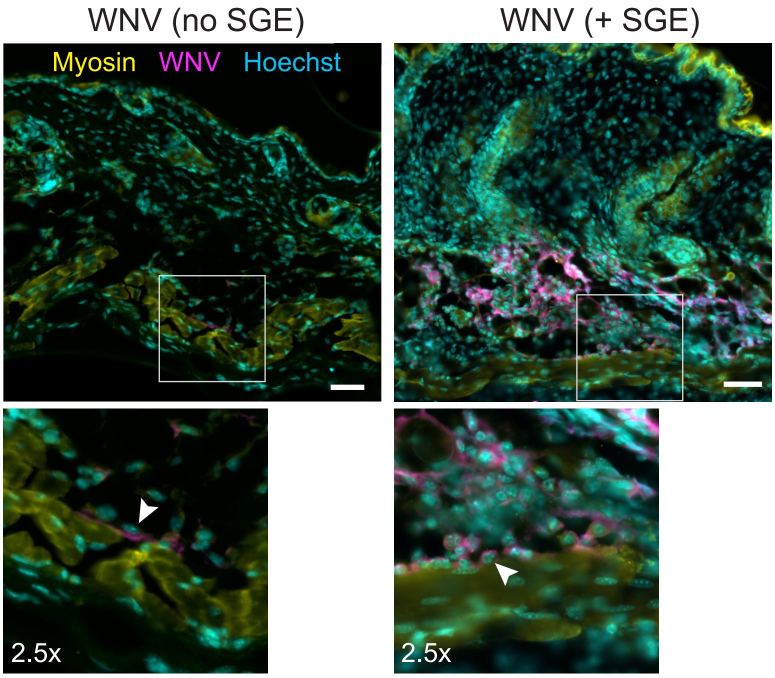

Figure S2- Intradermal WNV infection does not infect panniculus carnosus muscle cells even in the presence of *Aedes aegypti* salivary gland extract. Weanling C57BL/6 mice were injected with West Nile virus intradermally in the back skin with (right panel) and without (left panel) SGE. Immunofluorescence staining was performed for myosin (yellow), pan-flavivirus surface protein (magenta), and nuclei using Hoechst dye (cyan). Arrowheads indicate WNV-positive cells adjacent to the muscle layer, but not infection within muscle cells. Skin from SGE injected animals exhibits dermal thickening resulting from immune activation and infiltration (right panels) and was consistent across animals in that group. n = 4 mice per group with 2 sections stained per mouse with similar results. Scale bars = 50 μm.

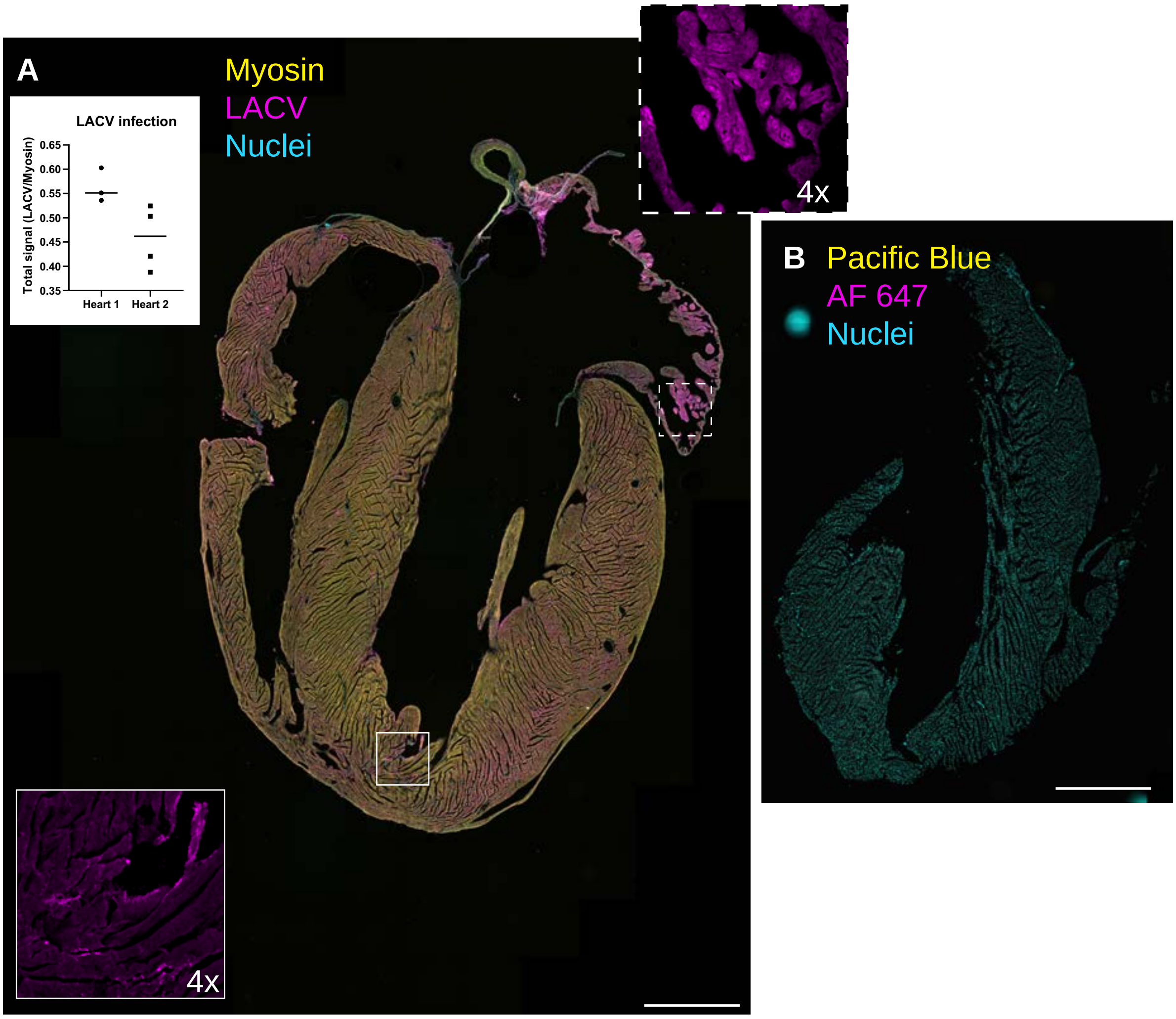

Figure S3: Heart muscle cells are infected with LACV during ID infection.
Heart tissue from ID infected mice at 6 dpi (A) was sectioned and stained for myosin (yellow), LACV (magenta), and Hoechst (cyan). Additional sections from the same mice were stained with Hoechst and the matching secondary antibodies to (A) without primary antibodies as a control (B). n = 2 mice with 3-4 heart sections stained per animal with similar results. Scale bar represent 1000 μm with insets at 4x zoom. LACV total signal intensity was quantified relative to myosin signal for two independent hearts across 3 (heart 1) or 4 (heart 2) separate sections per heart. Source data for (A) are provided as a Source Data file.

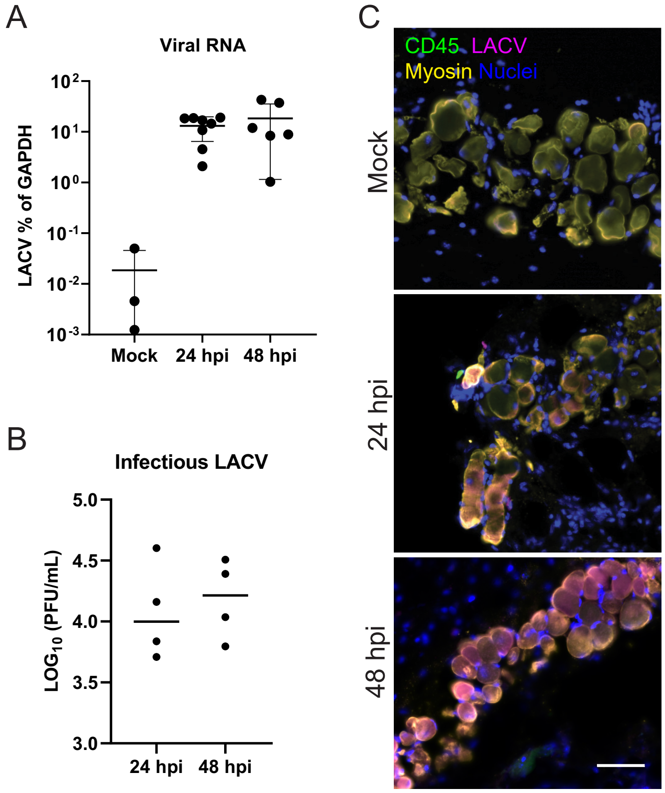

Figure S4: Ex vivo skin muscle can be isolated from the dermal immune cells and productively infected in culture. Panniculus carnosus muscle was excised from the skin of naive mice and infected *ex vivo* with LACV. RNA transcripts were quantified from the tissue by qPCR (A) or infectious virus quantified by plaque assay at 24 and 48 hpi (B) with each sample denoted by an individual symbol. Immunohistochemistry staining was also performed for the pan-immune cell maker CD45 (green) in addition to myosin (yellow) and LACV (magenta) across the time course with nuclei labeled with Hoechst (blue) (C). n = 3-8 skin muscle pieces per analysis (A,B) with each sample represented as a unique data point. For (C), images are representative across two independent experiments with similar results. Scale bar = 50 µm. Source data for (A,B) are provided as a Source Data file.

Table S1. Antibodies used for Immunofluorescence

Application	Antibody	Vendor (Catalognumber)	Dilution	Figure
Primary	Rabbit anti-LACV	Hyperimmune serum generated in house	1:400	1A-B, 1D; 3A-C, 3E-F; 4A (except brain)-4B; 5B, 5D-E; S3B; S4C
Primary	Mouse anti-LACV	Hyperimmune serum generated in house[1]	1:100	4A (brain)
Primary	Rabbit anti-active caspase 3 (biotin)	Cell signaling (9654S)	1:400	4A (except brain), 4B
Primary	Rabbit anti-active caspase 3	Promega (G748A)	1:250	4A (brain)
Primary	Mouse anti-myosin	Sigma-Aldrich (M1570)	1:300	1A-B, 1D; 3A-C, 3F; 5D; S3B; S4C
Primary	Rabbit anti-myosin	Millipore (476126)	1:200	S2
Primary	Chicken anti-GFP	Aves Labs (GFP-1020)	1:500	1A, D; 3A-C; 4B;
Primary	Mouse anti-GM130	BD Biosciences (610822)	1:400	5B, 5E
Primary	Rat anti-CD90	Biolegend (105202)	1:250	1B
Primary	Mouse anti-pan flavivirus (4G2)	Millipore (MAB10216)	1:100	S2
Conjugated primary	Rat Anti-CD45-FITC	BD Pharmingen (553079)	1:100	S4C
Secondary	Donkey anti-Rabbit AF594	Invitrogen (A21207)	1:500	4A (brain); S2
Secondary	Donkey anti-Rabbit AF647	Invitrogen (A31573)	1:500	1A-B, D; 3A-C, 3E-F; 4A (except brain)-4B; 5B, 5D-E; S3B; S4C
Secondary	Goat anti-mouse Pacific Blue	Invitrogen (P31582)	1:500	1A-B, 3A-C
Secondary	Donkey anti-mouse AF 488	Invitrogen (A21202)	1:500	5B, 5E;
Secondary	Donkey anti-mouse AF594	Invitrogen (A21203)	1:500	1D; 3F; 5D; S3B; S4C
Secondary	Donkey anti-mouse AF647	Invitrogen (A31571)	1:500	4A (brain); S2
Secondary	Donkey anti-chicken AF488	Invitrogen (A11039)	1:500	1A, D; 3A-C; 4A-B;
Secondary	Streptavidin DyLight 405	Invitrogen (21831)	1:500	4A (except brain), B
Secondary	Steptavidin AF594	Invitrogen (S32356)	1:500	4B
Secondary	Goat anti-rat AF488	Invitrogen (A11006)	1:400	1B; S4C

1 Evans, A. B., Winkler, C. W. & Peterson, K. E. Differences in Neuropathogenesis of Encephalitic California Serogroup Viruses. *Emerg Infect Dis* **25**, 728-738, doi:10.3201/eid2504.181016 (2019).
