# Supplementary material for: Skin muscle is the initial site of viral replication for arboviral bunyavirus infection
Source: Nat Commun. 2024 Feb 6;15:1121. doi: 10.1038/s41467-024-45304-0 (PMC10847502; doi:10.1038/s41467-024-45304-0)
Supplement: Supplementary file 3 — Reporting Summary [file 41467_2024_45304_MOESM3_ESM.pdf]

nature portfolio

Corresponding author(s): Karin E. Peterson

Last updated by author(s): Jan 11, 2024

Reporting Summary

Nature Portfolio wishes to improve the reproducibility of the work that we publish. This form provides structure for consistency and transparency in reporting. For further information on Nature Portfolio policies, see our Editorial Policies and the Editorial Policy Checklist.

Statistics

For all statistical analyses, confirm that the following items are present in the figure legend, table legend, main text, or Methods section.

n/a	Confirmed	
☐	☒	The exact sample size (n) for each experimental group/condition, given as a discrete number and unit of measurement
☐	☒	A statement on whether measurements were taken from distinct samples or whether the same sample was measured repeatedly
☐	☒	The statistical test(s) used AND whether they are one- or two-sided<br>*Only common tests should be described solely by name; describe more complex techniques in the Methods section.*
☒	☐	A description of all covariates tested
☒	☐	A description of any assumptions or corrections, such as tests of normality and adjustment for multiple comparisons
☐	☒	A full description of the statistical parameters including central tendency (e.g. means) or other basic estimates (e.g. regression coefficient) AND variation (e.g. standard deviation) or associated estimates of uncertainty (e.g. confidence intervals)
☐	☒	For null hypothesis testing, the test statistic (e.g. F, t, r) with confidence intervals, effect sizes, degrees of freedom and P value noted<br>*Give P values as exact values whenever suitable.*
☒	☐	For Bayesian analysis, information on the choice of priors and Markov chain Monte Carlo settings
☒	☐	For hierarchical and complex designs, identification of the appropriate level for tests and full reporting of outcomes
☒	☐	Estimates of effect sizes (e.g. Cohen's d, Pearson's r), indicating how they were calculated

Our web collection on statistics for biologists contains articles on many of the points above.

Software and code

Policy information about availability of computer code

Data collection	Data collection was done using Zen Black or Zen Blue (For confocal and slide scanning microscopy), QuantStudio Real-Time PCR software (in a QuantStudio 6 Flex machine, Applied Biosystems), and Gen5 software (for BioTek optical plate reader, Agilent)
Data analysis	Graph pad Prism (9.3.1), Microsoft Excel (2010), Imaris (9.9.1) specifically the Spots and Surface modules, Zen Blue (1.1.2.0), and FIJI/ImageJ (1.53t)

For manuscripts utilizing custom algorithms or software that are central to the research but not yet described in published literature, software must be made available to editors and reviewers. We strongly encourage code deposition in a community repository (e.g. GitHub). See the Nature Portfolio guidelines for submitting code & software for further information.

Data

Policy information about availability of data

All manuscripts must include a data availability statement. This statement should provide the following information, where applicable:

- Accession codes, unique identifiers, or web links for publicly available datasets
- A description of any restrictions on data availability
- For clinical datasets or third party data, please ensure that the statement adheres to our policy

The data used and/or analyzed during the current study are presented in this study and included in the supplemental files. Additional images are available from the corresponding author upon request.

Research involving human participants, their data, or biological material

Policy information about studies with human participants or human data. See also policy information about sex, gender (identity/presentation), and sexual orientation and race, ethnicity and racism.

Reporting on sex and gender	This research does not involve humans
Reporting on race, ethnicity, or other socially relevant groupings	This research does not involve humans
Population characteristics	This research does not involve humans
Recruitment	This research does not involve humans
Ethics oversight	This research does not involve humans

Note that full information on the approval of the study protocol must also be provided in the manuscript.

Field-specific reporting

Please select the one below that is the best fit for your research. If you are not sure, read the appropriate sections before making your selection.

[x] Life sciences [ ] Behavioural & social sciences [ ] Ecological, evolutionary & environmental sciences

For a reference copy of the document with all sections, see nature.com/documents/nr-reporting-summary-flat.pdf

Life sciences study design

All studies must disclose on these points even when the disclosure is negative.

Sample size	Sample sizes were determined by GraphPad Statmate for biological replicates of in vivo work using power analysis. For all in vitro experiments, sample size was not calculated. However, based on previous experience with our cell culture system all experiments were set up with triplicate samples with repeated experiments. This provides controls for both in-experiment variation and between-experiment variation. The sample sizes are described in each figure legend and the appropriate number to perform the appropriate statistical testing.
Data exclusions	No data was excluded
Replication	All experiments involved at least 2 biological replicates (for some imaging experiments) but often ≥3 biological replicates as detailed in the manuscript. For animal experiments, all data comes from at least 2 independent cohorts. Each mouse is represented as an individual data point on plots to add greater clarity to how many samples were processed per time point. For NSC and SMEV quantification, 5 images were quantified and each individual cell represented by an individual data point. All attempts at replication of results was successful for each experiment.
Randomization	No formal statistical methods were employed for randomization. Mice randomly assigned to cages by third party technicians and cells/tissues were analyzed equally with no sub-sampling, negating the need for randomization. Mice were allocated to time points across cages within a biological replicate.
Blinding	Infections and downstream analysis of mouse tissues were all done by the same investigator, thus blinding was not possible for allocation of mice to individual groups. However, tissue removed from animals were given a designated number and group information was not present on vials, tubes or sections until data was collected and analysis completed. Additionally, for Golgi analysis in NSCs and muscle cells was performed computationally, with the same thresholding applied to the LACV signal to all samples to minimize potential for human bias.

Reporting for specific materials, systems and methods

We require information from authors about some types of materials, experimental systems and methods used in many studies. Here, indicate whether each material, system or method listed is relevant to your study. If you are not sure if a list item applies to your research, read the appropriate section before selecting a response.

Materials & experimental systems

n/a	Involved in the study
☐	☒ Antibodies
☐	☒ Eukaryotic cell lines
☒	☐ Palaeontology and archaeology
☐	☒ Animals and other organisms
☒	☐ Clinical data
☒	☐ Dual use research of concern
☒	☐ Plants

Methods

n/a	Involved in the study
☒	☐ ChIP-seq
☒	☐ Flow cytometry
☒	☐ MRI-based neuroimaging

Antibodies

Antibodies used

1) Rabbit anti-LACV Hyperimmune serum generated in house.
2) Mouse anti-LACV Hyperimmune serum generated in house.
3) Rabbit anti-active caspase 3 (biotin). Cell signaling (#9654S, clone 5A1E| Lot# 2)
4) Rabbit anti-active caspase 3. Promega (#G748A, clone 5A1E| Lot# 0000155356)
5) Mouse anti-myosin. Sigma-Aldrich (#M1570, clone MY-32|Batch# 0000128667)
6) Rabbit anti-myosin. Millipore (#476126, polyclonal | Lot# 3772227)
7) Chicken anti-GFP Aves Labs (#GFP-1020, polyclonal| Lot # GFP3717982)
8) Mouse anti-GM130 BD Biosciences (#610822, clone 35/GM130| Lot# 9140853)
9) Rat anti-CD90. Biolegend (#105202. clone G7| Lot# B305481)
10) Mouse anti-pan flavivirus (4G2). Millipore (#MAB10216, clone D1-4G2-4-15 | Lot# Not available)
11) Rat anti-CD45-FITC. BD Pharmingen (#553079, clone 30-F11 | Lot # M070096)

12) Donkey anti-Rabbit AF 594 Invitrogen (#A21207, Polyclonal) | Lot# 2066086)
13) Donkey anti-Rabbit AF 647 Invitrogen (#A31573, Polyclonal) | Lot# 1626613)
14) Goat anti-mouse PacificBlue Invitrogen (#P31582, Polyclonal) | Lot# 796015)
15) Donkey anti-mouse AF 488 Invitrogen (#A21202, Polyclonal)| Lot# 2266877)
16) Donkey anti-mouse AF 594 Invitrogen (#A21203, Polyclonal)| Lot# 1608644)
17) Donkey anti-mouse AF 647 Invitrogen (#A31571, Polyclonal) | Lot# 1900251)
18) Donkey anti-chicken AF 488 Invitrogen (#A11039, Polyclonal)| Lot# 2304258)
19) Streptavidin DyLight 405 Invitrogen (#21831, Polyclonal)| Lot# Not available)
20) Streptavidin AF 594 Invitrogen (#S32356, Polyclonal) | Lot# 2273779)
21) Goat anti-rat AF 488 Invitrogen (#A11006, Polyclonal) | Lot# 34745A)

Validation

For all primary antibody staining a no-primary control was included for every experiment to validate specificity of staining and prevent interpretation of background staining as a false positive.

1-2. Anti-LACV antibody is validated in several prior studies in Dr. Peterson's lab (Winkler et al, Acta Neropath, 2015, Ojha et al., Nat. Micro., 2021, Basu et al, J. Neuroinflamm., 2021). A mock control is also stained alongside infected tissue to ensure there is no non-specific antibody staining.
3. Manufacturer validated the antibody in multiple tissues in embryonic tissues including citing localization in the correct location as well as extensive citations using the antibody. See (https://www.cellsignal.com/products/primary-antibodies/cleaved-caspase-3-asp175-5a1e-rabbit-mab/9664).
4. This product is discontinued and the manufacturer supplied website of specifications is no longer available. However, over 87 publications have used this antibody for IHC and ICC purposes successfully https://www.citeab.com/antibodies/7602042-g7481-anti-active-caspase-3-pab. Our lab has routinely used this antibody to demonstrate cell death in virus infected cells, with nearly complete absence of any staining in mock-infected mice, as expected.
5. Manufacturer mentions "Monoclonal Anti-Skeletal Myosin may be used for staining of human, rabbit, rat, mouse, bovine, chicken and guinea pig skeletal myosin. The antibody localizes an epitope on the myosin chain that is stable to the routine formalin-fixation and paraffin-embedding process"https://www.sigmaaldrich.com/US/en/product/sigma/m4276.
6. Manufacturer mentions "This Anti-Myosin Rabbit pAb is validated for use in Immunoblotting, Immunofluorescence, Frozen Sections, Immunoprecipitation for the detection of Myosin."https://www.emdmillipore.com/US/en/product/Anti-Myosin-Rabbit-pAb,EMD_BIO-476126.
7. Manufacturer mentions "Antibodies were analyzed by western blot analysis (1:5000 dilution) and immunohistochemistry (1:500 dilution) using transgenic mice expressing the GFP gene product." https://www.aveslabs.com/products/anti-green-fluorescent-protein-antibody-gfp
8. The 35/GM130 monoclonal antibody recognizes GM130, regardless of phosphorylation status.https://www.bdbiosciences.com/en-us/products/reagents/microscopy-imaging-reagents/immunofluorescence-reagents/purified-mouse-anti-gm130.610822.
9. Manufacturer validated the antibody for frozen IHC with mouse spleen sections. https://www.biolegend.com/nl-be/products/purified-anti-mouse-cd90-antibody-244.
10. Per the manufacturer "Anti-Flavivirus Group Antigen Antibody, clone D1-4G2-4-15 detects level of Flavivirus Group Antigen & has been published & validated for use in IF". https://www.emdmillipore.com/US/en/product/Anti-Flavivirus-Group-Antigen-Antibody-clone-D1-4G2-4-15,MM_NF-MAB10216
11. Manufacturer routinely validates the antibody by flow cytometry of mouse splenocytes. We validated the antibody in IHC by confirming labeling of CCR2-RFP cells in the skin in addition to numerous cells in the epidermis and dermis consistent with immune

cell morphology and location. The antibody did not stain myosin+ cells.

For 12-21, these are all secondary antibodies independently validated by Invitrogen with this statement "Specificity of secondary antibody was demonstrated by specific detection of the target immunoglobulin". In all cases, western blot was used to show band size is correct as well as extensive immunofluorescence staining demonstrating specificity. For this study, we always included a no primary control tissue in each staining to ensure that there is we do not detect non-specific binding due to the secondary antibodies. In all experiments, no secondary antibody non-specific binding was observed.

Eukaryotic cell lines

Policy information about cell lines and Sex and Gender in Research

Cell line source(s)	1. H9 human embryonic stem cell. Thermo Fisher Scientific -Invitrogen. derived human neural stem cells (hNSCs) (Catalog nos. N7800-100)<br>2. Vero cells. ATCC® CRL-1587<br>3. M007 human immortalized muscle cell line derived from a female healthy human donor (under IRB protocol #STUDY00000409 from the University of Minnesota)<br>4. F007 human immortalized fibroblast cell line derived from the same female healthy human donor as M007 (under IRB protocol #STUDY00000409 from the University of Minnesota).
Authentication	Vero cells were used only for plaque assays and virus propagation and were not authenticated. H9 cells were validated to express neural markers previously in the Peterson lab. M007 and F007 were validated by Dr. Kyba's lab after initial isolation and immortalization using morphology, cell sorting for CD56, and karyotyping to confirm 46,xx.
Mycoplasma contamination	Regular mycoplasma testing is not performed on these cell lines.
Commonly misidentified lines<br>(See ICLAC register)	None used

Animals and other research organisms

Policy information about studies involving animals; ARRIVE guidelines recommended for reporting animal research, and Sex and Gender in Research

Laboratory animals	Mus musculus, wild-type C57BL/6J and Ccr2RFP/+ Cx3cr1GFP/+, male or female, infected when between 20-23 days old (aka weanlings). Ccr2RFP/+ Cx3cr1GFP/+ heterozygous mice were generated by crossing Ccr2RFP/RFP mice (Jax strain 017586) with Cx3cr1GFP/GFP mice (Jax strain 005582). HET mice and C57BL/6J mice were bred in-house and used after weaning at 20-23 days post birth.<br>Aedes aegypti mosquitoes (liverpool strain, LVP) were used at 5-7 days old for salivary gland isolation.
Wild animals	The study did not involve wild animals
Reporting on sex	Both sexes were used in these studies with no sex-related phenotype observed. Males are denoted on plots by filled circles and females as open circles.
Field-collected samples	The study did not involve samples collected from the field
Ethics oversight	All animal experiments were approved by the Rocky Mountain Laboratories Animal Care and Use Committee.

Note that full information on the approval of the study protocol must also be provided in the manuscript.
